# Supplementary material for: The effect of repeat length on Marcal1-dependent single-strand annealing in Drosophila
Source: Genetics. 2022 Oct 27;223(1):iyac164. doi: 10.1093/genetics/iyac164 (PMC9836020; doi:10.1093/genetics/iyac164)
Supplement: iyac164_Supplementary_Data [file iyac164_supplementary_data.docx]

Supplemental Figures for Dewey *et al.*

**Figure S1**. Efficiency of I-*Sce*I*­* cutting. A region spanning the I-*Sce*I*­* cut site was amplified by PCR from red-eyed progeny, then subjected to cutting with I-*Sce*I*­*. If amplified DNA is unable to be cut by I-*Sce*I*­ in vitro*, we concluded that repair involved canonical NHEJ that mutated the cut site. An intact I-*Sce*I*­* indicates either failure to cut or precise repair *in vivo*. (A) Analysis of red-eyed SSA PCR products across all homologies (excluding 50 bp, as there were no red-eyed progeny) Overall, the percent of uncut products from red-eyed progeny is not significantly higher in wild type than in *Marcal1* mutants (*p*=0.0930, Fisher’s Exact test). (B) Analysis of red-eyed SSA PCR products for the 3.5 kb homology. The percent of uncut products from red-eyed progeny is not significantly higher in wt than in *Marcal1* mutants for the 3.5 kb homology (*p*=0.1383, Fisher’s Exact test). (C) Analy­sis of red-eyed SSA PCR products for the 2 kb 5’ and 3’ homologies. The percent of uncut products from red-eyed progeny is not significantly higher in wild type than in *Marcal1* mutants for the 2 kb homologies (*p*=0.6456 for 2 kb 5’; *p*>0.9999 for 2 kb 3’, Fisher’s Exact test). (D) Analysis of red-eyed SSA PCR products for the 500 bp homologies. The percentage uncut pro­ducts from red-eyed progeny is not signi­fi­cantly higher in wild type than in *Marcal1* mutants for both the 500 bp 5’ and 500 bp 3’ homologies (*p*=0.6514 for 500 bp 5’, *p*=0.3242 for 500 bp 3’, Fisher’s Exact test).

**Supplemental Figure S2**. To determine whether maternal contribution of Marcal1 might account for residual SSA in *Marcal1* homozygous mutant males, we compared SSA frequencies from these males (+/-, referring to the genotype of their mothers) to males whose mothers were homozygous for the *Marcal1* mutation (-/-). There were no significant differences in any experiment. *p*=0.2623 for 3.5 kb; 0.2689 for 2 kb 5’; 0.3112 for 500 bp 5’; 0.7715 for 50 bp 3’, unpaired t-test for each.

**Supplemental Figure S3**. Molecular analysis of repair products in white-eyed progeny from 50 bp 3’ assay. The percentage of SSA repair in white-eyed progeny for the 50 bp 3’ homology in wild type is not significantly different from that in *Marcal1* mutants (*p*=0.6015, Fisher’s exact test).


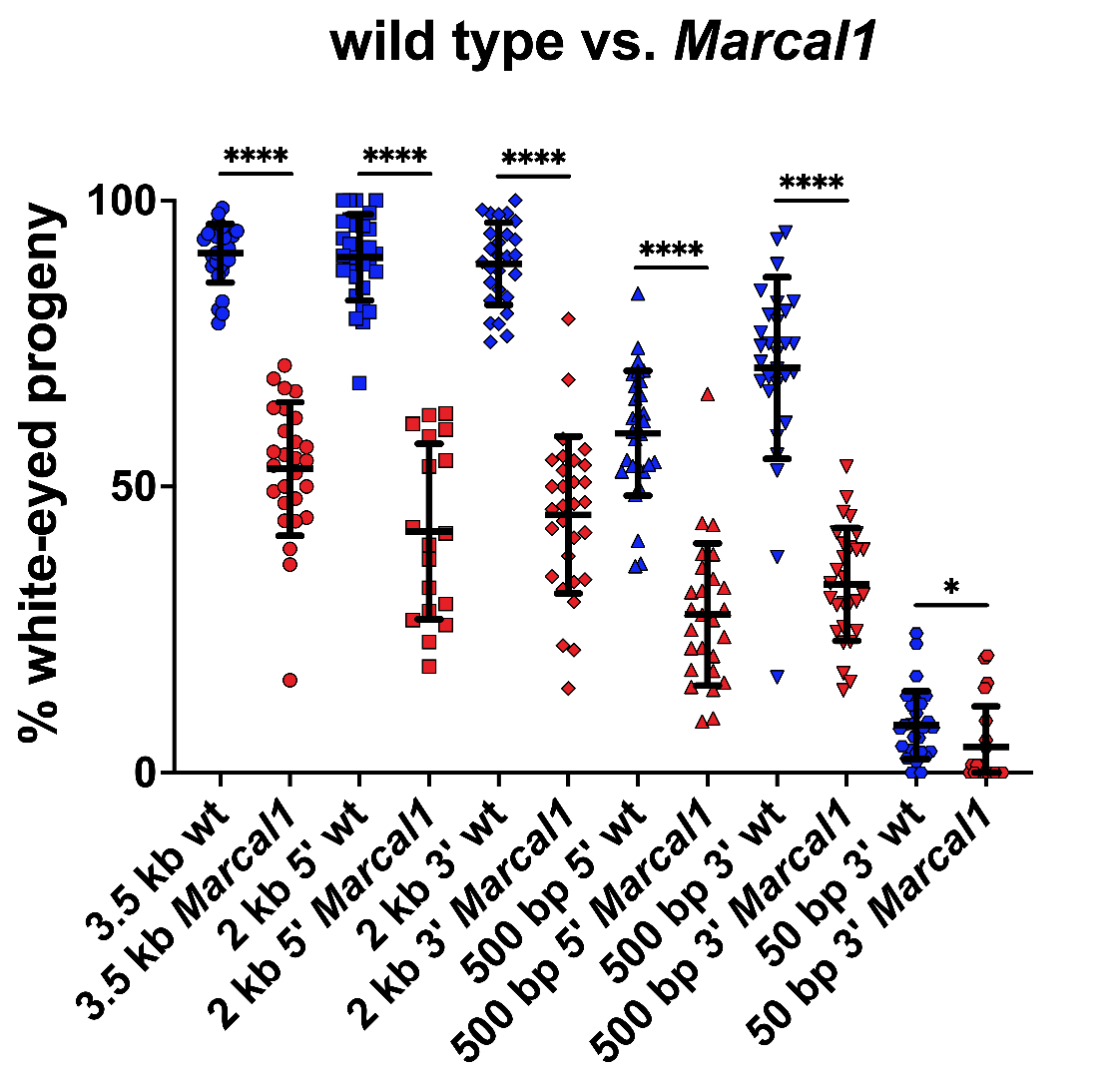
**Supplemental Figure S4.** Comparison between percent of white-eyed progeny for each homology for wild type (wt) versus *Marcal1* mutants. *****p* <0.0001 for 3.5 kb; *****p* <0.0001 for 2 kb 5’; *****p* <0.0001 for 2 kb 3’; *****p* <0.0001 for 500 bp 5’; *****p* <0.0001 for 500 bp 3’; **p* <0.05 for 50 bp 3’, unpaired t-test for each.
